# Reward processing in autism: a thematic series

**DOI:** 10.1186/1866-1955-4-20

**Published:** 2012-07-19

**Authors:** Gabriel Dichter, Ralph Adolphs

**Affiliations:** 1 Department of Psychiatry and the Carolina Institute for Developmental Disabilities, University of North Carolina at Chapel Hill, Chapel Hill, USA; 2 Departments of Psychology and Neuroscience and Biology, California Institute of Technology, Pasadena, CA, USA

## Abstract

This thematic series presents theoretical and empirical papers focused on understanding autism from the perspective of reward processing deficits. Although the core symptoms of autism have not traditionally been conceptualized with respect to altered reward-based processes, it is clear that brain reward circuitry plays a critical role in guiding social and nonsocial learning and behavior throughout development. Additionally, brain reward circuitry may respond to social sources of information in ways that are similar to responses to primary rewards, and recent clinical data consistently suggest abnormal behavioral and neurobiologic responses to rewards in autism. This thematic series presents empirical data and review papers that highlight the utility of considering autism from the perspective of reward processing deficits. Our hope is that this novel framework may further elucidate autism pathophysiology, with the ultimate goal of yielding novel insights with potential therapeutic implications.

## Review

This thematic series presents theoretical and empirical papers focused on understanding autism from the perspective of reward processing deficits. This framework suggests that the well-known deficits in social functioning that characterize individuals with autism may reflect or may even be caused by decreased motivation to interact with the social world and decreased feelings of pleasures during social exchanges (for example, [1-3]). Likewise, it may be the case that restricted and repetitive behaviors, another core feature of autism, may reflect altered reward-based processes: for example, individuals with autism demonstrate patterns of circumscribed interests, preoccupations and attachments that may reflect, at least in part, heightened interest, motivation, and pleasure in response to idiosyncratic objects [4,5].

Although the core symptoms of autism have not traditionally been conceptualized with respect to altered reward-based processes, it is clear that brain reward circuitry plays a critical role in guiding social and nonsocial learning and behavior throughout development [6]. Additionally, recent animal and nonclinical human data suggest that brain reward circuitry may respond to social sources of information in ways that are similar to responses to primary rewards [7]. Finally, as reviewed in this thematic series, recent clinical data consistently suggest abnormal behavioral and neurobiologic responses to rewards in autism. Together, these lines of evidence suggest that considering autism from the perspective of reward processing deficits may yield novel insights with potential therapeutic implications. Given that neuroscience-based investigations of motivational processes represents one of the domains specifically highlighted in the National Institute of Mental Health Research Domain Criteria initiative (http://www.nimh.nih.gov/research-funding/rdoc.shtml), we feel that understanding autism through the lens of reward systems has the potential to both improve etiologic understanding of autism and to suggest novel approaches for diagnosis and intervention.

Research on reward processing in autism is in a nascent stage, and there is a pressing need for empirical studies to define the boundary conditions of reward processing deficits in this disorder. This thematic series contains six empirical papers spanning a number of measurement methods. Cascio *et al.*[8] extend the functional magnetic resonance imaging literature on response to monetary, social and nonsocial object rewards by examining the neural mechanisms of response to appetizing food images in children with autism who had abstained from eating. To date, the framework for the so-called social motivation hypothesis of autism has been that children with autism may lack the motivation to participate in social behaviors where social skills are typically forged (evidenced by, for example, decreased orienting to social stimuli [1,9]). This results in an impoverished social environment that may further negatively impact the development of social skills [10,11]. By examining neural responses to food cues, Cascio and colleagues evaluated whether decreased motivation in autism extends to a primary reward cue. In their sample of patients with autism spectrum disorders, they found no evidence of diminished responses to food in the amygdala, nucleus accumbens, orbitofrontal cortex, or insula, and evidence of enhanced activation in the insula and anterior cingulate cortex. This finding dovetails with other data in the literature that reward processing deficits in autism may be domain-specific, rather than domain-general [12-16].

McPartland *et al.*[17] used event-related potentials to evaluate feedback-related negativity as well as early visual processing components in children with autism. The authors found no evidence of feedback-related negativity differences in autism during a guessing-game task. These data replicate and extend prior findings [18,19] by focusing on a younger sample and by contrasting gain responses with no-gain (rather than loss) responses. As such, this study represents an important replication and extension of prior electrophysiological studies of reward processing functions in autism, and suggests that reward processing deficits in autism may not extend to cortical responses to external and concrete error-related feedback.

Lin *et al.*[20] examined social preferences in autism by an analysis of behavioral patterns of donations to charities spanning categories pertaining to people, mental health, animals or the environment. The group of adults with high-functioning autism was found to donate relatively less overall, showed less discrimination in overall patterns of donating, and donated less to charities benefiting people relative to other categories, despite evidence that explicit preferences for specific charities did not differ between groups. This intriguing finding suggests a deficit in autism in translating intact social knowledge to behavioral choices linked to the value of social preferences.

Damiano *et al.*[21] used an effort-based decision making task, the Effort Expenditure for Rewards Task (EEfRT), to measure behavioral output of the mesolimbic dopamine system. The EEfRT was originally developed as a homologue of seminal preclinical tasks used to demonstrate the role of mesolimbic dopamine in effort-based decision-making [22]. These authors found that, across all levels of reward probability and magnitude, a high-functioning autism group selected harder task choices to obtain monetary rewards, suggesting decreased decision-making efficiency. Furthermore, across both diagnostic groups, harder task choices were correlated with cognitive manifestations of repetitive behaviors, suggesting a potential linkage between reward processing deficits and repetitive behavior symptoms.

This thematic series also includes a pupilometry study by Sepeta *et al.*[23] that adds to the growing body of evidence that certain social stimuli are less rewarding to individuals with autism. They present pupilometry evidence that children and adolescents with autism show a decreased reward response (that is, less increase in pupil diameter) to happy faces with direct gaze than do their neurotypical counterparts. This study is important not only for its substantive findings but also because it suggests a measure of reward processing deficits in autism that may be collected by stand-alone eye-trackers as well as by eye-trackers integrated within functional magnetic resonance imaging scanners.

This thematic series is complemented by a number of review papers as well. Kohls *et al.*[14] evaluate the empirical functional neuroimaging, electrophysiological and neurochemical literatures addressing reward system function in autism and conclude that, despite inconsistencies in this literature, a consistent picture is emerging of disrupted social reward ‘wanting’ capacities in autism. These authors suggest that this conclusion is consistent with the new diagnostic criteria for autism spectrum disorders that will be put forth in the Diagnostic and Statistical Manual, Fifth Edition that highlights deficits in spontaneous self-initiated seeking of social encounters, and they propose implications of this model for biobehavioral approaches to treatment. Future research addressing the temporal chronometry of reward responses in autism will be needed to more comprehensively confirm this model.

Dawson et al. [24]) review the literature on social attention impairments in autism and potential neural mechanisms underlying impaired social reward processing in autism. By stressing the linkage between social attention and social motivation, the authors connect the relatively new literature on reward processing in autism to the broader extant literature addressing social attention impairments in autism, including decreased orienting, attention and preferences for people, faces and speech sounds, and concomitant increased attention to nonsocial objects. They also suggest that the evolutionarily related nonapeptides oxytocin (OT) and vasopressin offer insights into the molecular and cellular mechanisms involved in reward processes supporting social behaviors given: (1) their roles as modulators of neural activity that regulate social behaviors and clinical evidence of reduced circulating levels of plasma OT in children with autism spectrum disorders linked to levels of social functioning; (2) the recent identification of the OT receptor (OXTR) as a susceptibility locus for autism; (3) evidence that variations in OXTR influences the development of social skills in autism; and (4) initial clinical trials suggesting that intranasal administration of OT improves social functioning in individuals with autism spectrum disorders.These authors additionally outline a framework by which measures of social attention may be used within the context of clinical trials as either mediators or moderators of response to behavioral or pharmacological interventions aimed at improving social functioning in autism.

Dichter *et al.*[4] take a broader approach to examining reward processing deficits in autism by reviewing the preclinical and clinical literatures addressing reward system dysfunction in a range of psychiatric and neurodevelopmental disorders, as well as providing brief overviews of effective psychopharmacologic agents that impact dopamine systems in these disorders. These authors outline the utility of identifying a potential common mechanistic process across disparate disorders, provide methodological considerations for research addressing reward circuitry functioning across disorders, and expose current gaps in knowledge about the functional integrity of reward processing systems across disorders.

Kishida *et al.*[25] describe recent advances in examining the neural basis of social exchange using economic games and the relevance of a social decision-making framework for understanding social impairments in autism. This neuroeconomics approach is built on foundational work that exchanges of money and social signals as well as of trust and cooperation are mediated by a largely overlapping neural circuitry, and urges the cognitive neuroscience community to co-opt the extant knowledge base addressing the brain bases for social exchange to better understand the pathophysiology of autism.

Finally, Watson *et al.*[26] provide a rich overview of animal studies addressing social reward motivation. This review discusses findings from studies of non-human primates and mice, and offers recommendations for motivational process that are suitable for study in each of these analogue models. In particular, the authors suggest that social interactions among primates are dynamic, complex, visual and include subtleties that make this model appropriate for understanding the brain basis of social motivation in humans. Additionally, they suggest that the deep homology in brain circuitry mediating social rewards in non-human primates is well-suited to addressing the neural basis of social motivation in humans.

## Conclusions

This thematic series suggests a novel framework for understanding the core features of autism, and, perhaps most critically, includes reviews that suggest the utility of considering this framework for understanding variability in treatment response in autism. Articles contained within this thematic series introduce a new theoretical perspective, review vast literature across several disciplines, and suggest ways to leverage an established neurobiological framework and associated behavioral and neuroimaging tasks for understanding the core features of autism. Our hope with this thematic series is to encourage other researchers to consider this framework, and to complement existing research programs addressing social-communicative deficits and restricted and repetitive behaviors in autism.

## **Authors’ Information**

GD is an Assistant Professor of Psychiatry and Psychology and an investigator at the Carolina Institute for Developmental Disabilities at the University of North Carolina at Chapel Hill. RA is the Bren Professor of Psychology and Neuroscience and a Professor of Biology at the California Institute of Technology.
